# Uncertainty quantification for patient-specific domain in virtual aortic procedures: application to thoracic endovascular aortic repair

**DOI:** 10.1007/s10237-025-02036-4

**Published:** 2025-12-14

**Authors:** Vittorio Lissoni, Anna Ramella, Giulia Luraghi, Puck Stassen, Wouter Huberts, Santi Trimarchi, Francesco Migliavacca, Jose Felix Rodriguez Matas

**Affiliations:** 1 Computational Biomechanics Laboratory–LaBS, Department of Chemistry, Materials and Chemical Engineering “Giulio Natta”, Politecnico di Milano, Piazza L. da Vinci 32, 20133 Milan, Italy; 2 Cardiovascular Biomechanics, Biomedical Engineering, Eindhoven University of Technology, Eindhoven, Netherlands; 3 Foundation IRCCS Ca’ Granda Ospedale Maggiore Policlinico, Milano, Italy; 4 Department of Clinical Sciences and Community Health, University of Milan, Milano, Italy

**Keywords:** In silico medicine, Digital twin in healthcare, Finite element analysis, VVUQ, TEVAR

## Abstract

Simulating medical procedures requires accounting for inherent uncertainty in many numerical model parameters, such as material properties. Evaluating the impact of these uncertainties is crucial for identifying parameters needing precise definition and correctly interpreting simulation results. This study explores how uncertainties in modelling the aorta affect finite element outcomes of a thoracic endovascular aortic repair (TEVAR) procedure. Based on literature data, aortic wall thickness and mechanical properties were identified as the most uncertain. The aorta was modelled using shell elements with homogeneous thickness and assumed to behave as a linear elastic isotropic material. A design of experiments approach was used for uncertainty quantification and sensitivity analysis: wall thickness and Young’s modulus were varied over 11 levels in a full factorial design, resulting in 121 simulations. Uncertainty was quantified using statistical metrics such as mean, standard deviation, coefficient of variation, and 95% confidence intervals. Results indicate wall thickness significantly affects aortic wall stress (σ_aorta_), with minimal influence on stent stress (σ_stent_) and device opening area (OA). Conversely, Young’s modulus has limited impact on σ_aorta_ but affects σ_stent_ and OA to a greater extent. The highest uncertainty was observed in σ_aorta_ (~ 25% coefficient of variation), while σ_stent_ and OA showed lower variability (2.6% and 6.9%, respectively). These findings suggest that, in this model, accurate wall thickness definition is more critical than precise Young’s modulus for reducing uncertainty in wall stress predictions. Therefore, literature-based averages for Young’s modulus may be sufficient for simulating this procedure.

## Introduction

Numerical simulations, particularly through the finite element method (FEM), have become an essential tool, providing powerful means for developing new medical devices and assisting surgeons in selecting the most appropriate device models and sizes (Mangado et al. 2016; Pathmanathan et al. 2019). Additionally, they help anticipate potential complications during surgical procedures. One notable application is the simulation of cardiovascular device implantation, such as the placement of the endograft in thoracic endovascular aortic repair (TEVAR), a minimally invasive procedure used to treat aortic pathologies, such as aneurysms and dissections. TEVAR has become the most adopted treatment since FDA approval in 2005 (Findeiss and Cody 2011; Nation and Wang 2015). However, implementing accurate simulations to be used in clinic is a delicate task. It requires continuous attention by the operator and insights into uncertainties in each phase of model development and execution. Uncertainties are introduced in all phases from segmenting patient-specific anatomies to assigning material properties and can, among others, be attributed to uncertainties due to the low accuracy and resolution of clinical data, or because certain parameters cannot be measured in vivo. These uncertainties can lead to significant errors in the results (Mangado et al. 2016; Pathmanathan et al. 2019). For instance, the limited resolution of medical imaging can impact the accuracy of vessel geometry used in simulations. Furthermore, many input parameters cannot be determined on a patient-specific basis (Liang and Mahadevan 2011). As a result, critical inputs such as material properties or boundary conditions are often approximated using average values derived from the literature.

To validate the model and enhance the interpretation of results, it is crucial to systematically assess both the sources and the extent of these uncertainties and their influence on simulation outcomes (Mangado et al. 2016; Pathmanathan et al. 2019). Uncertainty quantification (UQ) is a systematic approach to evaluate how these uncertainties affect simulation results, improving the robustness and reliability of numerical predictions (Mangado et al. 2016; Pathmanathan et al. 2019). UQ is particularly relevant in the FEM simulations of the TEVAR procedure, since even minor variations in input parameters can lead to significant discrepancies, potentially impacting clinical decisions. Uncertainties in the mechanical properties (i.e. Young’s modulus) of the aortic wall could affect stress/strain distribution or the correct positioning of the endograft. UQ is typically divided into two main components: uncertainty quantification and sensitivity analysis (SA) (Mangado et al. 2016; Pathmanathan et al. 2019). The first step involves quantifying input uncertainties and propagating them to the output by running multiple simulations, each time varying the uncertain input parameters, and subsequently characterizing the resulting output uncertainties. SA, on the other hand, aims to assess the relative influence of each uncertain input parameter on the final simulation results. This approach not only quantifies overall output uncertainty but also identifies the most influential input parameters, highlighting those that require more precise definitions to enhance simulation accuracy, while others can be fixed to population-based values (Eck et al. 2017).

In the literature, few studies in the cardiovascular field can be found that perform this kind of analysis, as most assume input data to be reliable; simulations are typically implemented with a fixed set of data carrying out a deterministic study with no focus on uncertainties. The main reason for this is related to the high computational costs and time associated with such analyses (Biehler and Wall 2018). The few examples available are recent, likely due to the increasing interest in the topic, and focus more on clinical applications than on device implantation or other surgical procedures (Biehler et al. 2015; Sankaran et al. 2016; Biehler and Wall 2018; Gheysen et al. 2024; Schäfer et al. 2024; Menon et al. 2024; Colebank and Chesler 2024; Keramat et al. 2024). To the best of our knowledge, no UQ study has yet been performed on the TEVAR procedure.

This study aims to investigate the uncertainties associated with aortic modelling in TEVAR simulations, focusing on identifying the primary sources of uncertainty and evaluating their impact on numerical results. Specifically, the uncertain parameters considered in this work are aortic wall thickness and its mechanical properties. These same sources of uncertainty were previously identified by (Gheysen et al. 2024) in their UQ analysis of pressurization in an idealized dissected aorta model. (Biehler and Wall 2018) investigated the effect of aneurysmatic wall thickness uncertainty on the risk of rupture. These studies showed a great influence of the wall thickness and a reduced influence of the mechanical properties on the resulting stresses and rupture risk predictions. Mechanical properties having a reduced effect on the stress state of vessels are also confirmed by the results in (Joldes et al. 2016; Liu et al. 2019; Dong et al. 2022), other than theoretical knowledge such as Laplace law. While these studies employed a hyperelastic model for the vessel behaviour, we adopted an isotropic linear elastic model to more clearly isolate and assess the impact of material stiffness uncertainty on simulation outcomes. Additionally, in all the cited works, the vessel was subjected to a homogeneous load without considering any procedure modelling or device interaction, as in the TEVAR simulation, for which an assessment of the effect of wall thickness and mechanical properties variability has still to be investigated. To perform a comprehensive uncertainty quantification of patient-specific TEVAR simulations, we employed a global UQ approach (Eck et al. 2017) using a design of experiments (DoE) methodology (Yoon et al. 2005). This involved systematically varying the uncertain input parameters across their entire uncertainty range—defined based on an extensive literature review—by sampling different values of both aortic thickness and Young’s modulus. The propagation of these uncertainties to the output was then evaluated by running simulations for all identified sample points. The selected quantities of interest were chosen to provide meaningful insights into the impact of these uncertainties on TEVAR procedure outcomes.

## Material and methods

### Clinical data and aortic modelling

The selected patient was a 63-year-old man who suffered from an asymptomatic penetrating ulceration (PAU) located in his left hemi-aortic arch. The patient had a bovine aortic arch with a common origin of the brachiocephalic trunk and left common carotid artery (LCCA). Diameters of the PAU measured 26 by 32 mm in axial and sagittal sections. The proximal stent-graft landing zone was directly distal to the bovine supra-aortic trunk (zone 2), and a 34 × 34 × 100 proximal FreeFlo Valiant Captivia® stent graft (Medtronic, Inc., MN, U.S.A.) was implanted. A 2-month control postoperative CTA (Computed tomography angiography) confirmed the thoracic aortic endograft’s correct position without complications. Approval for this specific study was waived by the local ethical committee (Ramella et al. 2023).

**Fig. 1:**
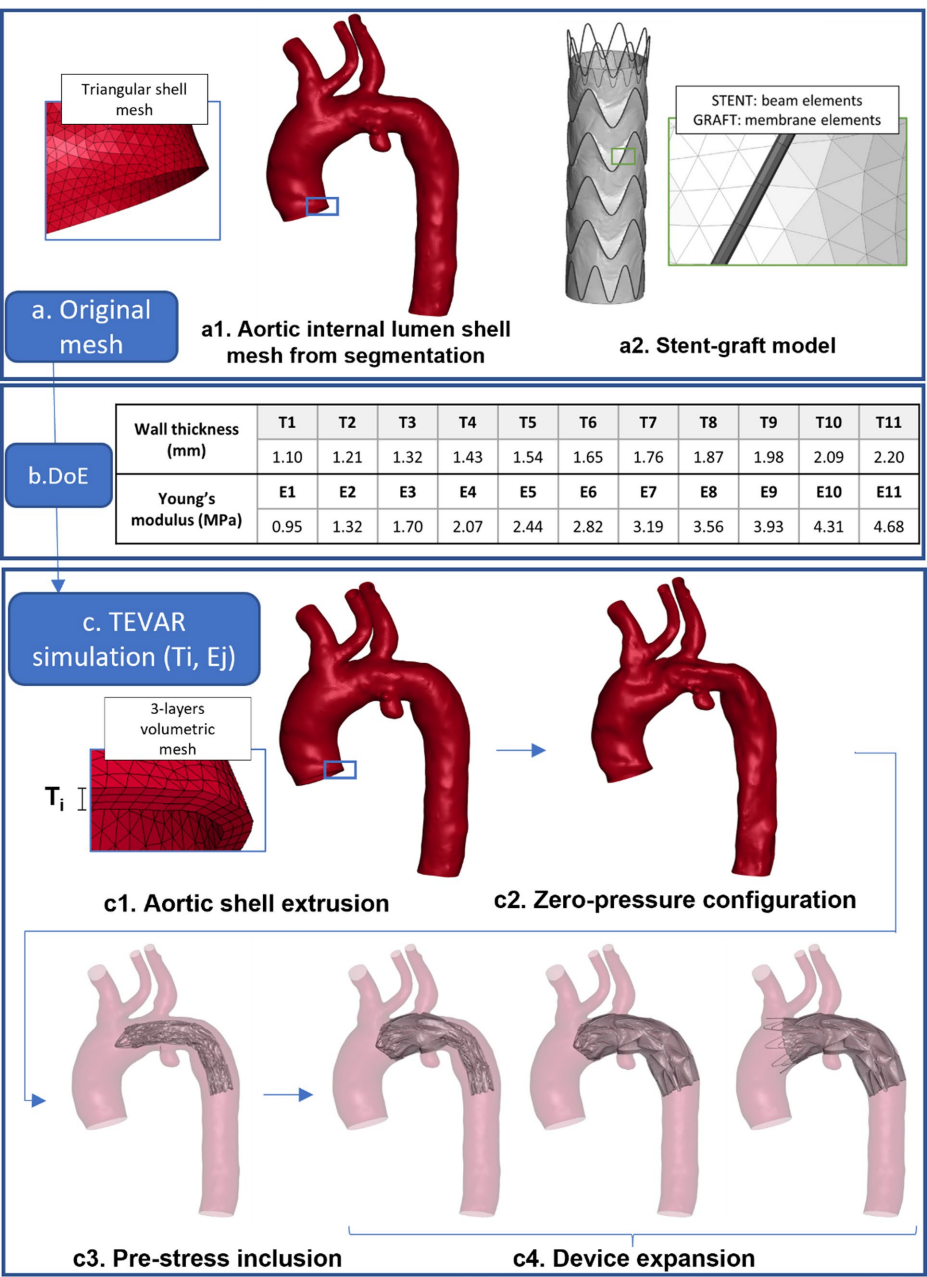
**a** Aortic and stent-graft mesh, **b** Design of Experiments, **c** workflow of the TEVAR simulation for the specific case with thickness Ti and Young’s modulus Ej

The lumen of the patient-specific aortic model (Fig. 1a) was segmented and pre-processed from pre-operative CTA images using VMTK (Vascular Modelling Toolkit, Orobix Srl) using a level-set technique. The obtained shell mesh of the internal lumen was extruded with tetrahedral elements to account for the thickness of the aorta (Fig. 1c1). A constant thickness is considered through the whole domain (Choudhury et al. 2009). An average element size of 0.75–1 mm was selected. The meshing procedure was carried out with ANSA Pre Processor v24.0 (BETA CAE System, Switzerland).

### Stent-graft numerical model

The stent-graft was modelled following (Ramella et al. 2023). The stent was discretized with beam elements (1232 elements, average size of 1 mm) and the graft with triangular membrane elements (16,414 elements, average size of 1 mm). Node-to-node connection between the two parts was imposed to take the presence of sutured points into account. Nitinol shape memory material formulation for the stent and a fabric material formulation with no resistance to compression for the graft were adopted. Nitinol and PTFE material properties assigned, respectively, to the stent and graft are reported in Table 1. Further material properties details can be found in (Ramella et al. 2022).

**Table 1:** Assigned Nitinol material properties

Stent	Density	ρ	0.00645 g/mm3
	Austenite Young’s Modulus	E_A_	57 500 MPa
	Austenite Poisson’s Ratio	ν	0.3
	Martensite Young’s Modulus	E_M_	47 800 MPa
	Martensite Poisson’s Ratio	ν	0.3
	Transformation strain	ε	0.063
	Start of Transformation Loading	σ ^S^_L_	550 MPa
	End of Transformation Loading	σ ^S^_L_	620 MPa
	Start of Transformation Unloading	σ ^S^_U_	450 MPa
	End of Transformation Unloading	σ ^E^_U_	250 MPa
	Start of transformation stress in compression	α	0.0279
Graft	Density	ρ	0.00137 g/mm^3^
	Young’s Modulus	E_G_	1060 MPa
	Poisson’s Ratio	ν	0.35

### Uncertainty quantification of input data and DoE

For a more straightforward interpretation of the results, a simplified aortic material model, already used in the literature, has been adopted, specifically an isotropic linear elastic (Di Martino et al. 2001; Li and Kleinstreuer 2005; Sturla et al. 2013; Rahmani et al. 2019). This work represents the first attempt in the literature to perform an uncertainty quantification analysis in the context of TEVAR simulations. For this reason, we consider the linear elastic assumption to be a reasonable and appropriate starting point for this initial investigation. Young’s modulus range was defined considering the values in these works and also, to extend the considered range of uncertainty, linearized Young’s modulus values from both the soft and stiffer regions of the hyperelastic curves in longitudinal and circumferential directions reported in the literature (Raghavan et al. 1996; Di Martino et al. 2001; Li and Kleinstreuer 2005; Scotti et al. 2005; Vande Geest et al. 2006; Azadani et al. 2012; Sommer et al. 2016; Xuan et al. 2021) were considered. The aorta was tested with a uniaxial tensile test both in longitudinal and circumferential directions in (Raghavan et al. 1996; Di Martino et al. 2001; Sommer et al. 2016) while planar biaxial tests were performed in (Vande Geest et al. 2006; Azadani et al. 2012; Xuan et al. 2021). The linearization was performed by considering the full stress–strain range before and after the transient region of the curve, corresponding respectively to the soft and stiffening regimes. The resulting minimum and maximum values were 0.95 MPa (Azadani et al. 2012) and 4.68 MPa (Raghavan et al. 1996), respectively. The density and Poisson’s ratio were assumed to be 1120 kg/m^3^ and 0.49, respectively (Ramella et al. 2023). Additionally, the aortic wall thickness was identified as another key source of uncertainty, arising in the segmentation process alongside the intrapatient variability (Vorp et al. 2003; Vande Geest et al. 2006; Azadani et al. 2012; Pasta et al. 2012; Prieto-González et al. 2012; Xuan et al. 2021). The minimum and maximum aortic wall thicknesses were 1.1 mm (Prieto-González et al. 2012) and 2.2 mm (Azadani et al. 2012).

In our model, wall thickness is assumed homogeneous across the entire vessel. For both Young’s modulus and wall thickness, statistical distributions were not available in the literature, which made it impossible to estimate an exact probability density function. Instead, their ranges were identified based on the minimum and maximum reported values, discretizing these ranges into uniform intervals. A global uncertainty quantification was implemented by combining each possible sample in the Design of Experiment (DoE). Specifically, each parameter was discretized into 11 evenly spaced steps, resulting in a total of 121 simulations. Figure 1b shows the discretized values for the two uncertain parameters that form the design of experiments.

### Uncertainty propagation through FEM simulations

In this analysis, the virtual TEVAR procedure developed by Ramella et al. (2023) and Ramella et al. (2022) is used. This method allows for simulating the TEVAR procedure on patient-specific anatomies, including the vessel pre-stress, which is the stress acting on the aorta at the moment of image acquisition due to internal blood pressure (Ramella et al. 2024). In the following paragraphs, the steps for implementing TEVAR simulations are outlined. These steps will be repeated for all the identified DoE combinations of aortic thickness and Young’s modulus.

#### Zero-pressure configuration

Before starting the TEVAR simulation, in the initial step, the aorta was deformed to its real undeformed shape, called zero-pressure configuration, following the methodology outlined in Ramella et al. 2024. To achieve this, an inverse elastostatic problem was solved in ANSYS Mechanical FEA software (Ansys Inc., Canonsburg, PA, USA) under the assumption of diastolic 80 mmHg acting at the moment of image acquisition. All nodes on the proximal and distal sections of the aorta and the sections of the supraortic vessels were constrained. The aorta was modelled as isotropic, incompressible, and linear elastic. From the resulting mesh, a triangular shell mesh of the internal lumen (Fig. 1c2) was extracted and will be used as the starting condition for the TEVAR simulation.

#### TEVAR simulation

FEM of the TEVAR simulation was performed on 28 CPUs of an Intel Xeon64 with 250 GB of RAM using the commercial explicit finite element solver LS-DYNA 971 Release 14.0 (ANSYS, Canonsburg, PA, USA). A selective mass scaling technique was adopted to maintain a constant time-step of 0.001 ms. In the first part of the simulation, the zero-pressure configuration of the aorta (calculated in 2.1.2) was pressurized to diastolic condition to account for the aortic pre-stress: a homogeneous load of 80 mmHg was applied to the vessel shell mesh (Fig. 1c3). After that, the stent-graft in its crimped configuration is moved along the vessel centreline to the diseased zone inside the guide catheter, modelled as a rigid body. Specifically, the device was delivered to the same landing zone as shown in the post-operative CTA image. From here, the penalty contact between the stent-graft and guide catheter was progressively released to allow the device to expand gradually, and the penalty contact between the aorta and the device began to ensure proper adaptation of the device to the vessel (Fig. 1c4). Once the device had completely adhered to the vessel, results were extracted. During the simulation, all nodes on the proximal and distal sections of the aorta and the sections of the supraortic vessels were constrained.

### Quantities of interest

A key quantity of interest was the stress distribution within the vessel, as it evaluates the interaction between the vessel and the device, in addition to serving as an estimator for complications such as aortic damage (Hemmler et al. 2019; Barati et al. 2022). In fact, focal high-stress regions have been found to strongly correlate with the locations of stent-graft-induced new entries in type B aortic dissections (Menichini et al.; Singh et al.), potentially compromising the device’s locking mechanism (Sengupta et al. 2023). The vessel wall stress was analysed at the most critical regions of the aorta corresponding to the vessel-device contact point in the aortic arch. Specifically, we extracted the highest von Mises stress from all simulations, which was consistently observed in the same regions of the aorta across all 121 simulations.

We identified the five areas where the highest von Mises stress is measured. The von Mises stress is then averaged across the element with the highest stress and all its neighbouring elements to filter out local fluctuations (σ_aorta_). Additionally, this study aimed to understand how uncertainties in the modelling of the aorta impact the behaviour of the stent-graft. To achieve this, we examined the opening area of each stent ring (OA) and the von Mises stress in the stent (σ_stent_). Again, we identified the three most stressed elements among the 121 simulations performed and extracted the stress for all of them. In this case, the stress was extracted from the element without averaging across surrounding elements due to the nature of the beam mesh. The uncertainties in the output data were investigated through a variance-based global uncertainty quantification analysis (Saltelli et al. 2008), using average value, standard deviation, coefficient of variation, and 95% confidence interval as measures. More specifically, a SA was carried out to further understand how each uncertain input data affects the output. The first-order Sobol sensitivity indices were calculated to assess the contribution of each input parameter to the total variance, and the second-order Sobol sensitivity indices were calculated to estimate the interplay of the two parameters on the total variance (Eck et al. 2017). In particular, the first-order Sobol sensitivity index is calculated as follows:

1 $$\begin{array}{c}{S}_{i}=\frac{V\left[E\left[Y|{X}_{i}\right]\right]}{V\left[Y\right]}\end{array}$$

The term $V[E[Y|{X}_{i}]$ represents the variance of the expected value of the generic output $Y$ fixing ${X}_{i}$ (either the wall thickness or Young’s modulus) and $V [Y]$ the total variance of the output $Y$. This index can be interpreted as the expected reduction in total output variance (uncertainty metric) that can be obtained when the parameter ${X}_{i}$ would be know exactly and thus provides insights into the parameter that is most rewarding to measure more accurately (Saltelli et al. 2008). For each possible value of ${X}_{i}$, we computed the average of the output $Y$ (i.e. the conditional mean), and then we calculated the variance of these averages.

The second-order sensitivity was calculated as:

2 $$\begin{array}{c}{S}_{ij}=\frac{V\left[E\left[Y|{X}_{i},{X}_{j}\right]\right]- V\left[E\left[Y|{X}_{i}\right]\right]- V\left[E\left[Y|{X}_{j}\right]\right]}{V\left[Y\right]}\end{array}$$

where ${X}_{i}$ and ${X}_{j}$ represent the wall thickness and the Young’s modulus, respectively, $V\left[E\left[Y|{X}_{i},{X}_{j}\right]\right]$ represents the variance of the output $Y$ by fixing both ${X}_{i}$ and ${X}_{j}$ values. In this case, since only two uncertain parameters are considered, the expected value of $Y$ fixing ${X}_{i}$ and ${X}_{j}$ coincides with the output data resulting from the simulation with ${X}_{i}$ and ${X}_{j}$. A dedicated sensitivity analysis, presented in Appendix A, was conducted to evaluate the reliability of the Sobol indices with the chosen number of 121 simulations.

## Results

**Fig. 2:**
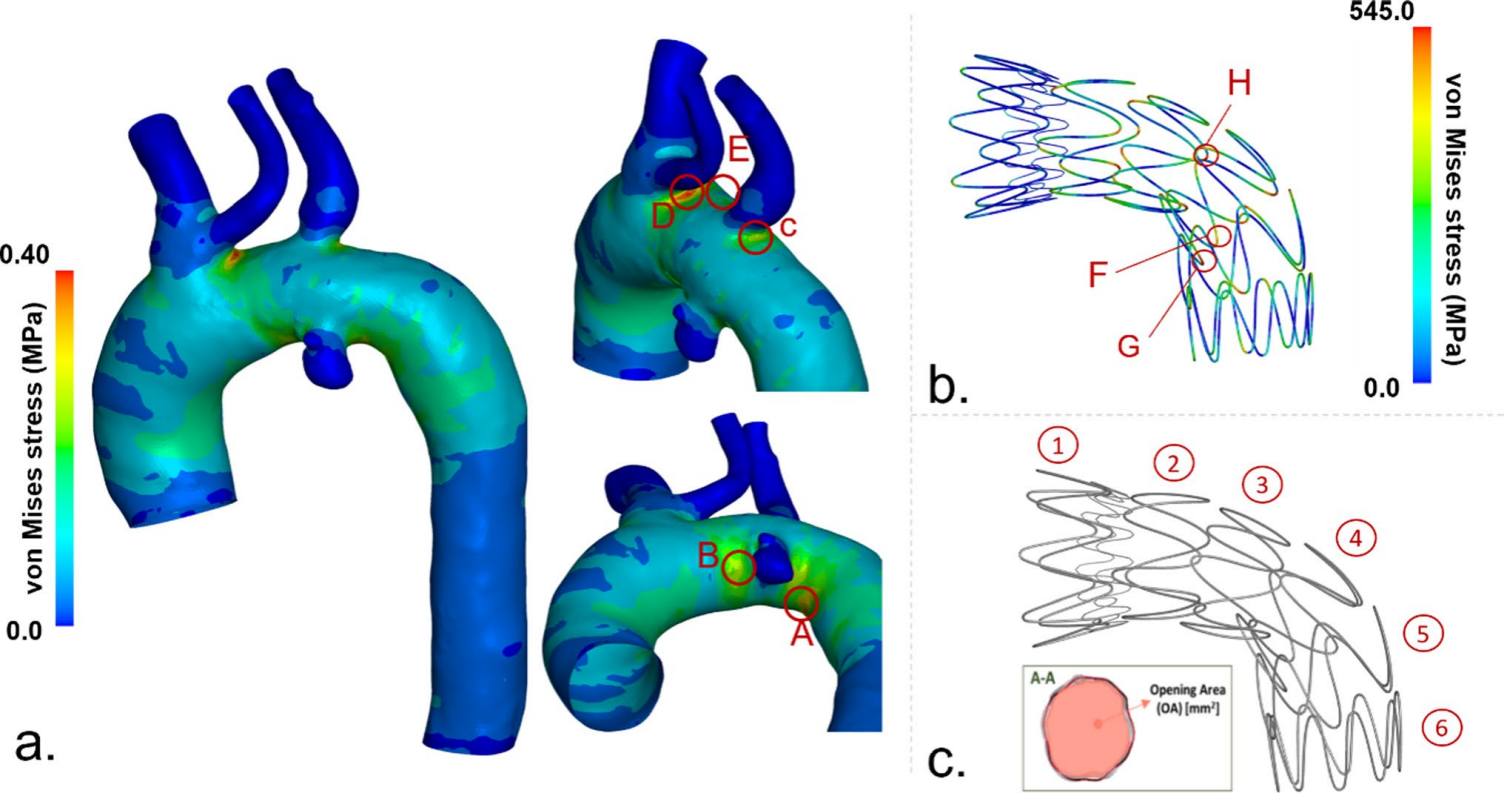
Quantities of interest; σ_aorta_ data were extracted from (**a**), σ_stent_ (**b**), and OA of each stent ring (**c**)

All the 121 simulations converged and were included in the uncertainty quantification analysis. The estimation of the zero-pressure configuration took approximately 5 min of computational time for each model, while TEVAR simulation took approximately 3 h for each model. In Fig. 2, the σ_aorta_ and σ_stent_ for the simulation with aortic wall thickness of 1.54 mm and Young’s modulus of 2.44 MPa are shown as an example. The maximal von Mises stresses in the aortic wall and stent are extracted from the regions indicated as A–G. A similar distribution was found for all other simulated cases, with peak values observed in the very same positions. The opening area is measured for each stent ring as shown in Fig. 2c.

In Table 2, average values, standard deviations, coefficients of variation, and 95% confidence intervals are reported for all quantities of interest.

**Table 2:** Results of uncertainty quantification for the quantities of interest investigated

		Average (MPa)	Standard deviation	Coefficient of variation	95% confidence interval
Aortic von Mises stress	Region A	0.379	0.0912	0.241	[0.363 0.400]
	Region B	0.305	0.0728	0.239	[0.292 0.318]
	Region C	0.337	0.0782	0.232	[0.323 0.351]
	Region D	0.371	0.0931	0.251	[0.354 0.388]
	Region E	0.336	0.0783	0.233	[0.322 0.350]
Stent von Mises stress	Element F	553	15.4	0.0278	[550 555]
	Element G	538	10.7	0.0199	[536 539]
	Element H	537	15.8	0.0295	[534 539]
Stent ring opening area	Stent ring 1	868	20.6	0.0237	[864 871]
	Stent ring 2	432	33.5	0.0776	[426 438]
	Stent ring 3	418	36.2	0.0865	[412 425]
	Stent ring 4	316	27.7	0.0878	[311 321]
	Stent ring 5	405	34.6	0.0854	[399 411]
	Stent ring 6	603	30.2	0.0500	[597 608]

### Sensitivity analysis

To perform SA, the quantities of interest were analysed separately by changing the values of the Young’s modulus and the wall thickness independently. The effect of each uncertain input parameter on the output was evaluated in terms of both first- and second-order Sobol indices, and in terms of average value and standard deviation for each level.

#### Aortic wall

**Fig. 3:**
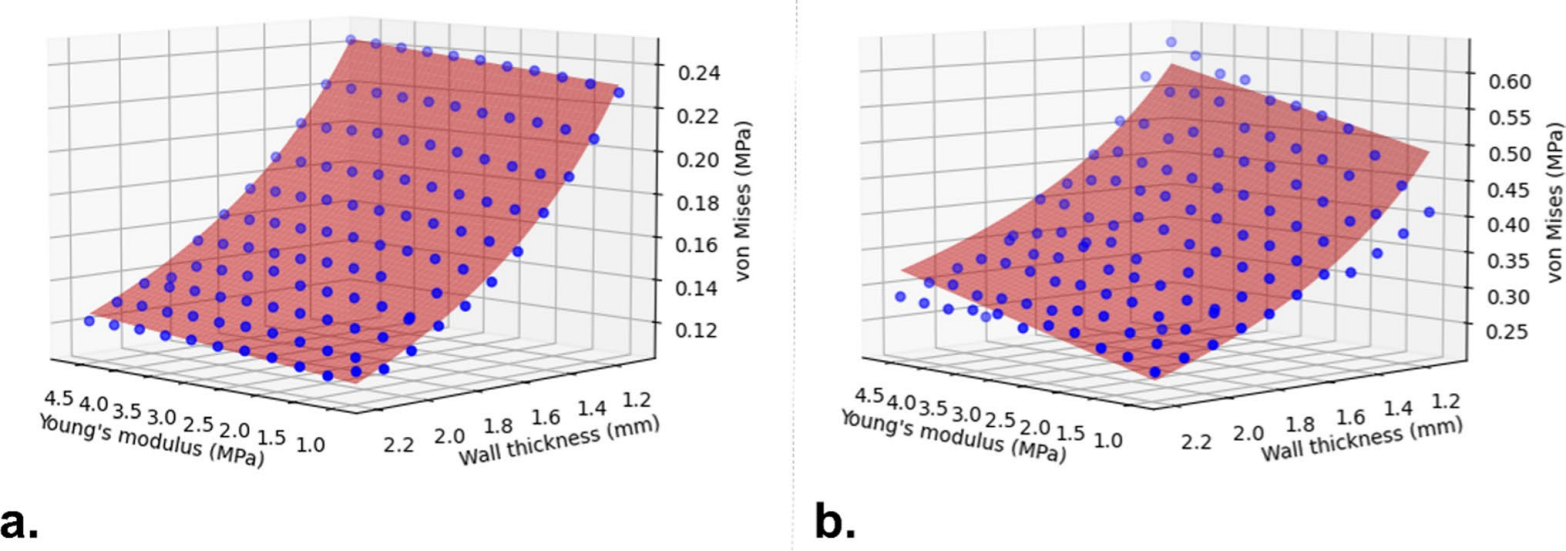
Fitting of the σ_aorta_ stress in region A (see Fig. 2) at the end of pre-stress inclusion (**a**) and at the end of the stent–graft release (**b**)

To assess the validity of our simulations, we fitted σ_aorta_ extracted at the end of the pressurization process (before the release of the stent) to the following function:

3 $$\begin{array}{c}\sigma =a+b*E+\frac{c}{T}\end{array}$$

where $E$ is the Young’s modulus, $T$ is the wall thickness, $a$, $b,$ and $c$ are coefficients. This aims to verify the ability of this model to simulate the physiological response of the vessel to an internal pressure described by the Laplace law:

4 $$\begin{array}{c}\sigma =\frac{P*r}{T}\end{array}$$

where $\sigma$ is the circumferential stress, $P$ is the pressure acting on the internal lumen, $r$ is the radius of the vessel and $T$ is the thickness. We obtained a very good fitting of the data confirmed by an R^2^ value of 0.996 (Fig. 3a). In particular, $b$ and $c$ resulted equal to 0.003 and 0.260, respectively, indicating a greater influence exerted by the wall thickness. Results in Fig. 3a allow to graphically visualize and better understand the distribution of output uncertainties due to selected input uncertainties only in response to aortic pressure.

**Fig. 4:**
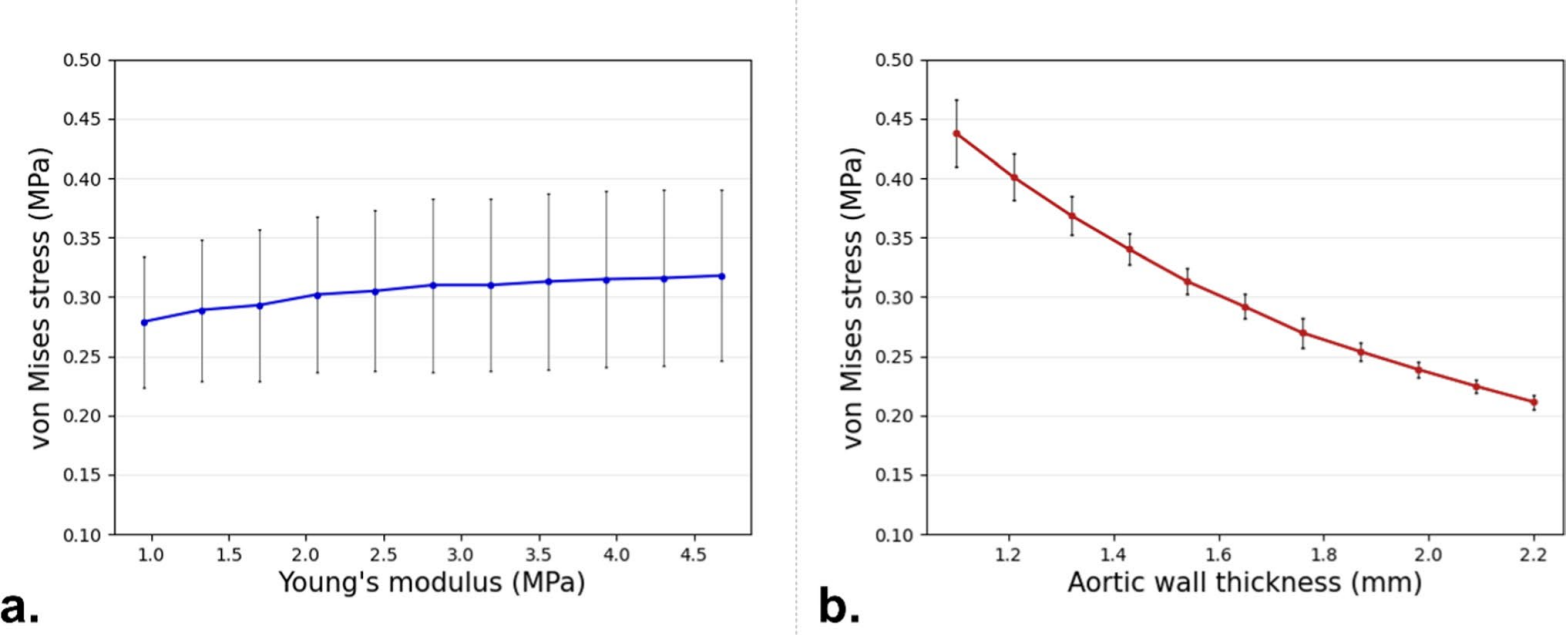
σ_aorta_ in aortic region B with respect to the Young’s modulus (**a**) and of the aortic wall thickness (**b**). Error bars displayed represent the standard deviation, indicating the variation caused by the wall thickness (**a**) and the Young’s modulus (**b**)

The relationship between σ_aorta_ in region B (region shown in Fig. 2a) in the aortic wall and Young’s modulus and wall thickness after the TEVAR procedure is depicted in Fig. 4. Figure 4a shows the positive correlation between σ_aorta_ and Young’s modulus of the aorta, with a high standard deviation indicated by the error bars, while Fig. 4b shows the effect of wall thickness on σ_aorta_. In this case, the stress decreases monotonically as wall thickness increases. This correlation shows smaller variability as compared to Young’s modulus, as indicated by the standard deviation in the error bars in Fig. 4.

To observe the influence of the two uncertain parameters more clearly, the model of Eq. 3 was fitted to values of σ_aorta_ at the end of the procedure (Fig. 3b). A good fit was assessed by an R^2^ value of 0.928. The greater influence of the wall thickness is also confirmed in this case (a = − 0.064, b = 0.026, c = 0.563).

**Fig. 5:**
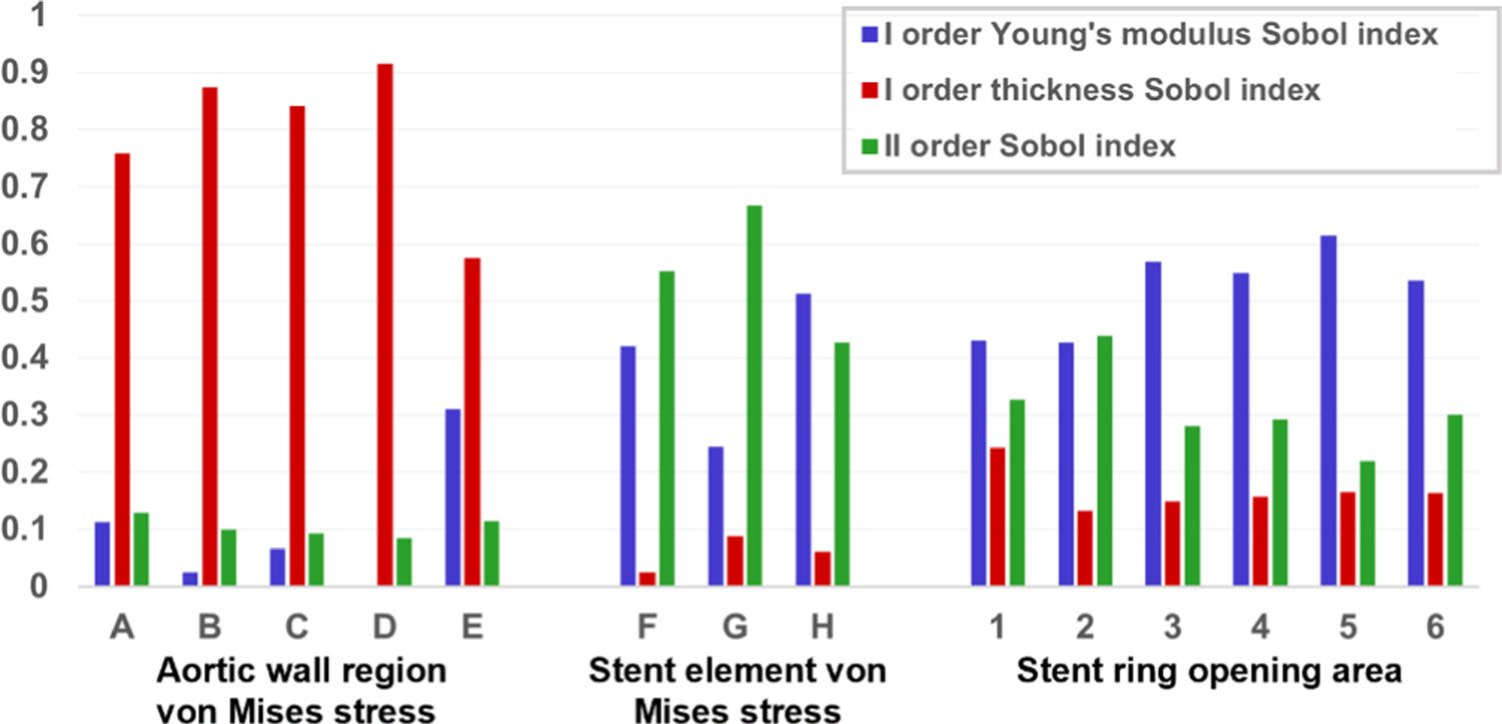
Bar chart of Sobol indices for the three quantities of interest

These results are confirmed by the first-order Sobol indices reported in Table 3 for region A, which are larger for wall thickness than for Young’s modulus. Regarding the second-order Sobol indices, the values are found to be consistently higher than the Young’s modulus Sobol index but still much lower than the wall thickness Sobol index. Similar results are found for regions B, C, D, and E, as shown in Fig. 5.

**Table 3:** σ_aorta_ stress SA results

	Young’s modulus first-order Sobol sensitivity index	Wall thickness first-order Sobol sensitivity index	Second-order Sobol sensitivity index
Wall region A	0.112	0.758	0.129
Wall region B	0.0257	0.874	0.0998
Wall region C	0.0657	0.841	0.0927
Wall region D	0.000185	0.915	0.0852
Wall region E	0.310	0.575	0.114

#### Stent graft

**Fig. 6:**
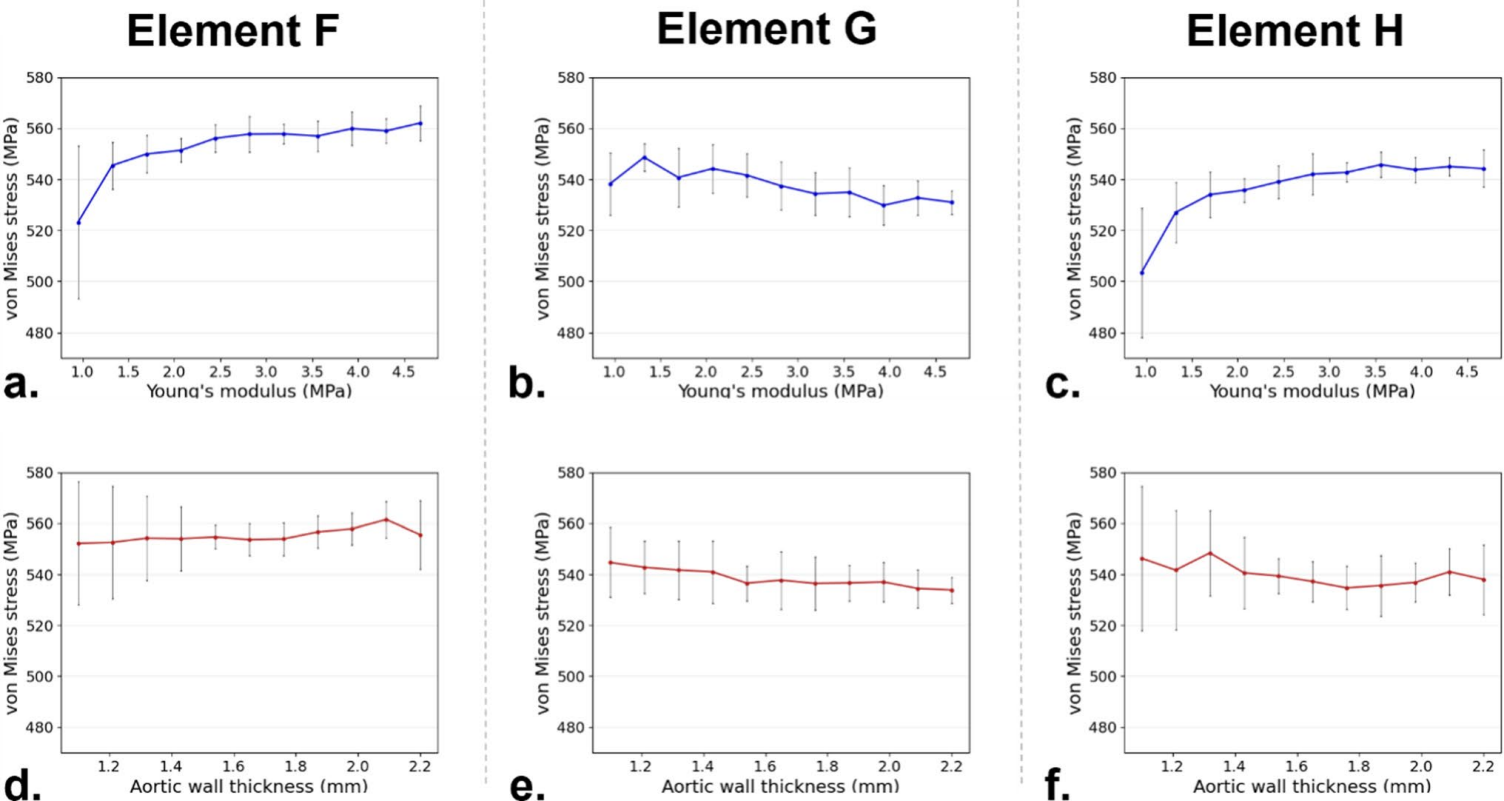
σ_stent_ with respect to Young’s modulus in element F (**a**), G (**b**), and H (**c**), and with respect to wall thickness in element F (**d**), G (**e**), and H (**f**)

For σ_stent_, the first-order Sobol sensitivity indices for the Young’s Modulus are larger than those for wall thickness, as shown in Table 4 and Fig. 5 (middle panel). The second-order Sobol index reaches high values, highlighting the importance of the interaction between two parameters in the stent response. For elements F and H, two stent elements in contact with the aortic wall, a limited increase in σ_stent_ is observed with increasing Young’s modulus (Fig. 6a and c).

**Table 4:** σ_stent_ stress SA results

	Young’s modulus first-order Sobol sensitivity index	Wall thickness first-order Sobol sensitivity index	Second-order Sobol sensitivity index
Stent element F	0.421	0.0253	0.553
Stent element G	0.245	0.0873	0.668
Stent element H	0.513	0.061	0.427

The limited effect of wall thickness on σ_stent_ is confirmed in Fig. 6d–f where the average σ_stent_ is not particularly influenced by increasing wall thickness. In general, the standard deviation decreases with Young’s modulus and wall thickness. Figures 6b and e show σ_stent_ for element G, which is not in direct contact with the aortic wall. Here, a decrease in stress is observed with both increasing E and thickness. Both graphs display a consistently large standard deviation.

#### Opening area

In Table 5 and Fig. 5 (right panel), it can be seen that the Sobol sensitivity indices of OA are higher for the aortic wall Young’s Modulus than for aortic wall thickness. Again, the second-order Sobol indices show higher values than the first-order ones for wall thickness, highlighting the interplay between these two uncertain input parameters.

**Table 5:** Opening area of the stent rings SA results

	Young’s modulus first-order Sobol sensitivity index	Wall thickness first-order Sobol sensitivity index	Second-order Sobol sensitivity index
Opening area 1	0.430	0.242	0.327
Opening area 2	0.428	0.133	0.439
Opening area 3	0.569	0.149	0.281
Opening area 4	0.550	0.157	0.292
Opening area 5	0.615	0.165	0.220
Opening area 6	0.536	0.164	0.300

**Fig. 7:**
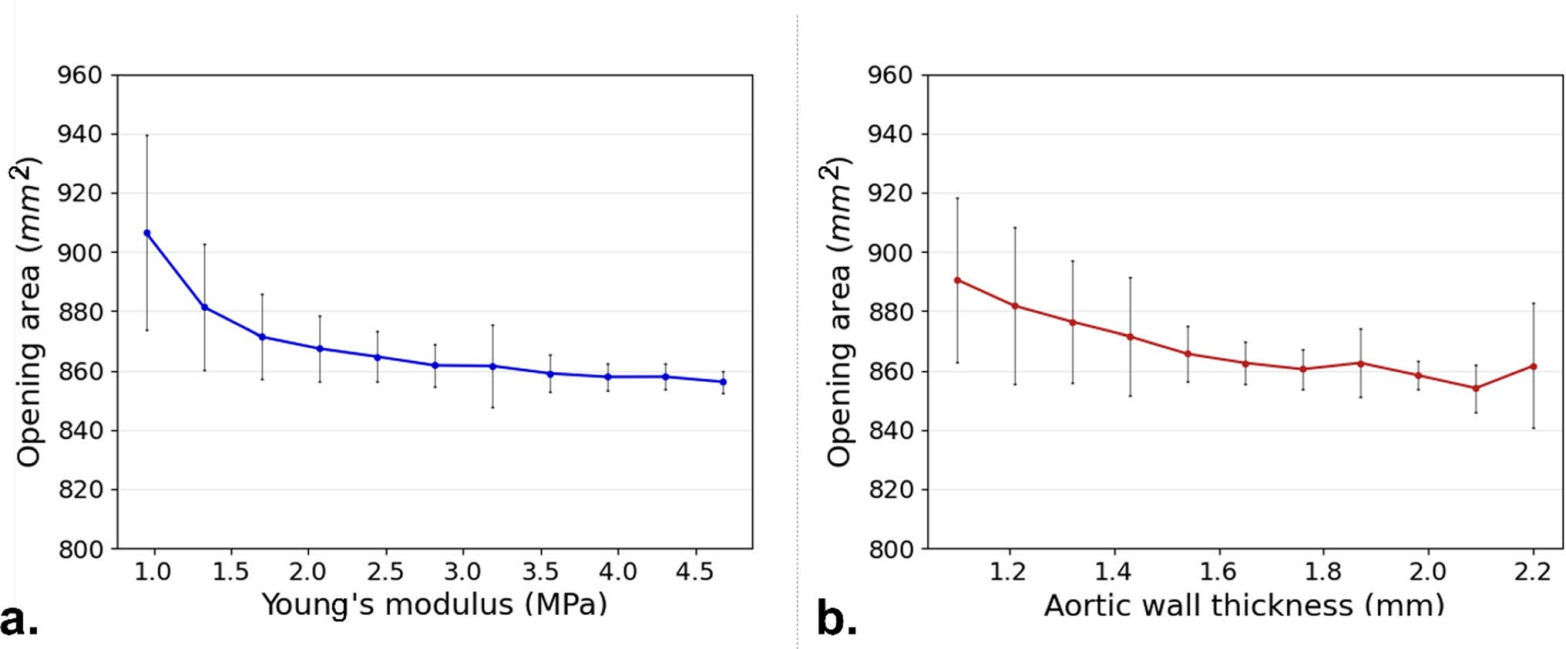
Average stent ring OA for stent ring 1 with respect to Young’s modulus (**a**) and wall thickness (**b**). the displayed error bars represent the standard deviation caused by either wall thickness (**a**) and Young’s modulus (**b**)

The relationship between OA and the two parameters of the first stent ring is shown in Fig. 7. Figure 7a demonstrates that as Young’s Modulus of the aorta increases, OA decreases. The trend stabilizes at higher values of E, where further increases in stiffness result in minimal changes to the OA. Figure 7b shows the effect of wall thickness on the OA. Similarly, as the wall thickness increases, the OA generally decreases. It can be noted that the left graph has a smaller standard deviation compared to the right. The other five stent rings displayed comparable relations and are not shown.

## Discussion

In this study, we performed a variance-based uncertainty quantification and global SA focused on the aortic wall modelling used in TEVAR simulations. Specifically, we considered two key uncertain parameters: the Young’s modulus and the wall thickness of the aorta. The uncertainty ranges for these quantities were defined based on values reported in the literature, capturing the physiological variability observed in both experimental and clinical data. Uncertainty propagation was carried out through a simulation workflow that has been previously validated (Ramella et al. 2023), ensuring that the resulting UQ and SA outcomes are based on a robust and reliable computational setup. This is essential for interpreting the impact of input variability within the context of the aortic model implemented. Regarding sensitivity analysis, we employed a global SA approach, which, unlike local methods, accounts for input variability across the full parameter space and captures interaction effects between variables. Importantly, the conclusions drawn from this analysis, regarding the influence of parameter uncertainty on the mechanical behaviour of the simulated aorta, must be understood in relation to the specific assumptions and structure of the modelling framework. We assessed the uncertainties in the quantities of interest σ_aorta,_ σ_stent_, and OA, in terms of mean, standard deviation, coefficient of variation, and 95% confidence interval. Overall, the uncertainty quantification results indicate that variations in wall thickness and material properties have a greater influence on the vessel’s state of stress, and so with the consequent device interaction, than on the stent-graft behaviour. Specifically, the coefficient of variation reached approximately 25.0% for σ_aorta_, while it remained below 9.0% and 3.0% for OA and σ_stent_ values, respectively. These findings are further supported by the 95% confidence intervals, which were wider for aortic stress and narrower for the other two parameters. Despite a significant increase—over fourfold in Young’s modulus and twofold in wall thickness—the uncertainty in OA remained limited. This suggests that the device expansion does not cause significant vessel deformation. Instead, the stent adapts to the vessel shape independently of the vessel’s mechanical properties. As a result, stent deformations remain largely unaffected, leading to stress values that are not widely dispersed around the mean. However, as stated previously, these results must be interpreted in terms of the characteristics of the device used in this study. A device with a different geometry or radial force to diameter behaviour could result in different values of the uncertainty quantities.

A more detailed uncertainty analysis through sensitivity evaluation further clarifies these results. Regarding the SA of σ_aorta_, wall thickness emerges as the most influential parameter affecting output, as confirmed by the first-order Sobol index in Table 2. This is also evident in Fig. 4b, where doubling wall thickness leads to a non-linear halving of σ_aorta_, accompanied by a limited and progressively decreasing standard deviation. Thicker walls distribute forces over a larger volume, reducing localized σ_aorta_ concentrations. This inverse relationship between stress and wall thickness aligns with Laplace’s law for cylindrical structures (Eq. 4). This relationship was further validated through data fitting in Fig. 3. A strong fit was observed after pre-stress inclusion and as well as at the end of device expansion. However, in the latter case, a lower R^2^ value suggests that numerical fluctuations from the aorta–stent contact algorithm are influencing the results. These results, observed prior to the device release, are consistent with previous findings in the literature, which determined that aortic wall stress, caused solely by internal pressure, does not change significantly with varying mechanical properties (Joldes et al. 2016; Liu et al. 2019; Dong et al. 2022). Sobol indices for Young’s modulus show significantly lower values, with second-order Sobol indices exceeding first-order indices. This indicates that Young’s modulus alone has a minor impact on σ_aorta_ variability but amplifies the effect of wall thickness uncertainty. Figure 4 illustrates the positive correlation between σ_aorta_ and Young’s modulus; yet, the high standard deviation highlights the substantial impact of wall thickness uncertainty. In addition, the second-order Sobol index for the aortic wall stress (see Fig. 5 left panel) suggests that prioritizing an accurate definition of vessel wall thickness is more critical than refining the material properties for which averaged values could be used. This is encouraging because patient-specific aortic wall thickness can be more easily measured from medical images, whereas characterizing patient-specific material properties remains challenging. This conclusion aligns with the findings of Gheysen et al. 2024 and J. Biehler and Wall 2018, who used a hyperelastic vessel model and arrived at similar results regarding aortic stress uncertainty.

In the stent, within the limited range of σ_stent_ uncertainty, Young’s modulus has a greater influence compared to wall thickness. Figure 6 illustrates that σ_stent_ increases with Young’s modulus, whereas the effect of wall thickness is minimal. Sobol sensitivity indices further confirm this, showing lower values for wall thickness. Additionally, the second-order Sobol index is consistently higher than its first-order counterpart for the wall thickness, indicating that while wall thickness alone may not play a dominant role in stent stress, it contributes to the overall vessel rigidity, amplifying the effect of Young’s modulus. The standard deviation in Fig. 6 is particularly high at lower Young’s modulus values, where vessel rigidity is lower. Interestingly, results for the two stent elements in contact with the aorta (elements F and H in Fig. 6) are consistent. However, for the element not in contact with the vessel, both Young’s modulus and wall thickness appear to reduce stress levels. Additionally, this element exhibits a larger standard deviation for both parameters, suggesting that its behaviour cannot be reliably predicted based on wall thickness and Young’s modulus alone. Lower first-order Sobol indices and higher second-order indices reinforce this, indicating that the interaction between uncertain parameters plays a dominant role. The interplay between Young’s modulus and wall thickness in determining overall vessel rigidity—with a stronger effect from the Young’s modulus—is also evident in OA results. Here, the first-order Sobol index for Young’s modulus and the second-order Sobol index are higher than the first-order index for wall thickness. As expected, increased vessel stiffness reduces stent rings OA, and the uncertainty in wall thickness has a greater impact at lower Young’s modulus values, as seen in the much higher standard deviation (Fig. 7). Overall, the analysis suggests that carefully selecting Young’s modulus could help mitigate uncertainty in predicting device behaviour more effectively than refining the definition of wall thickness. Notably, the largest dispersion in OA values remains below 9.0% around the mean, despite significant uncertainty in Young’s modulus (which increased more than fourfold in our study). This implies that using literature-based averaged values for Young’s modulus, rather than patient-specific ones, could still provide a reasonable representation of device response during TEVAR procedures.

This study is not without limitations. First, we employed an isotropic linear elastic model for the aorta, despite substantial evidence in the literature suggesting that a hyperelastic formulation would be more appropriate for its constitutive modelling (Holzapfel et al. 2000; Kamenskiy et al. 2014). The choice of a simpler model was intentional, as it allowed for a clearer interpretation of the effects of material properties without the additional complexity introduced by the non-linear dependence of hyperelastic parameters. Moreover, to our knowledge, this is the first attempt to perform an uncertainty quantification analysis in the context of TEVAR simulations. For this reason, we consider the isotropic linear elastic assumption a reasonable starting point; future work could extend the analysis to anisotropic and non-linear material models (Kamenskiy et al. 2014) in order to capture uncertainties related to hyperelastic parameters. Another limitation is that our analysis was conducted on a single patient-specific anatomy. Future studies should include more patients to explore whether anatomical variations influence the observed trends and to assess whether these findings hold across different patient populations. Lastly, another limitation arises from the choice of modelling the aorta with a constant thickness throughout the vessel. Because this type of analysis requires a large number of simulations, the use of shell elements enabled us to reduce the computational time. Although it is possible to prescribe variable thickness with shell elements, we opted to keep it constant in order to reduce the total number of variables and further reduce the computational burden. More refined simulations, aimed at surgical planning or at investigating potential complications, should instead incorporate patient-specific and location-specific thickness information.

## Conclusion

With this work, we intended to investigate how uncertainties in the material properties of the aorta would affect the results of patient-specific TEVAR simulations. Based on existing literature, we identified the range of the primary sources of uncertainty as the aortic wall thickness and Young’s modulus, although no clear statistical distribution was available. The aortic response was analysed by examining the von Mises stresses in the most solicited regions of the vessel, which are more likely to be subjected to complications such as aortic rupture. The device behaviour was assessed in terms of stent von Mises stress, focusing on the most solicited elements, and the stent ring opening area. The largest uncertainty was found in the wall stress, with the wall thickness being the most influential parameter. We believe that further attention to its characterization would be beneficial in reducing the uncertainties in this output. In contrast, the uncertainties in the two output parameters for the stent-graft appear to be influenced more by the Young’s modulus and are overall much less pronounced.

## Figures and Tables

**Fig. 8:**
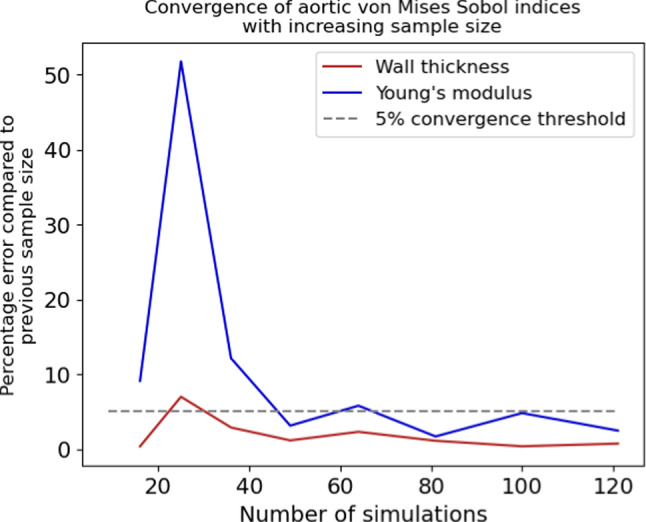
Results of the sensitivity analysis for aortic von Mises Sobol indices in region A

## Data Availability

No datasets were generated or analysed during the current study.

## Appendix A

A sensitivity analysis on the first-order Sobol indices of σ_aorta_​ in region A was performed to assess their stability and reliability, using a total of 121 simulations. Random sampling of discretized levels of wall thickness and Young’s modulus was conducted, progressively increasing the number of levels included in order to generate the simulation sets for the sensitivity analysis. Sobol indices were computed for each set and compared in terms of **percentage difference** between consecutive steps, defined as:

$$\text{Percentual difference}= 100 . \left|\frac{S({n}_{i})-S({n}_{i-1})}{{S(n}_{i})}\right|$$

The results demonstrate that the Sobol indices converge to within a 5.0% threshold already with 64 simulations. Detailed results are presented in Table 6 and Fig. 8.

**Table 6:** Results of the sensitivity analysis for von Mises stress in region A

N_simulazioni	Sobol_S	Percentual difference	Sobol_E	Percentual difference
9	0.781	–	0.16	–
16	0.779	0.359	0.176	9.09
25	0.837	6.97	0.116	51.7
36	0.814	2.87	0.132	12.1
49	0.824	1.15	0.128	3.13
64	0.805	2.30	0.121	5.79
81	0.814	1.11	0.119	1.68
100	0.817	0.367	0.125	4.80
121	0.823	0.729	0.122	2.46
